# Feasibility, safety and pharmacokinetic study of hepatic administration of drug-eluting beads loaded with irinotecan (DEBIRI) followed by intravenous administration of irinotecan in a porcine model

**DOI:** 10.1007/s10856-012-4768-2

**Published:** 2012-09-27

**Authors:** Andrew L. Lewis, Rachel R. Holden, S. Ting Chung, Peter Czuczman, Timothy Kuchel, John Finnie, Susan Porter, David Foster

**Affiliations:** 1 Drug Delivery Division, Biocompatibles UK Ltd, Farnham Business Park, Weydon Lane, Farnham, Surrey, GU9 8QL UK; 2 Institute of Medicine and Veternary Science, 101 Blacks Road, Gilles Plains, Adelaide, SA 5086 Australia

## Abstract

Irinotecan eluting embolization beads (DEBIRI) are currently being evaluated in the clinic for the treatment of colorectal cancer metastases to the liver. The aim of this study was to determine the safety and pharmacokinetics associated with two cycles of hepatic embolization using DEBIRI followed by intravenous administration of irinotecan. Pigs were embolized with DEBIRI (100–300 μm, 100 mg dose, *n* = 6) and blood samples taken over 24 h to determine plasma levels of irinotecan and SN-38 metabolite and for haematology and biochemistry. At 24 h an IV infusion of 250 mg/m^2^ of irinotecan was administered and the plasma levels taken again. This cycle was repeated 3 weeks later. A single animal was subjected to a more aggressive regimen of embolization with 200 mg bead dose and IV of 350 mg/m^2^ for two cycles. Three animals were sacrificed at 6 weeks and the remaining four (*n* = 3 standard dose, *n* = 1 high dose) animals at 12 weeks and detailed histopathology performed. All animals tolerated the treatments well, with only minor changes in haematological and biochemical parameters. There was no overlap in drug plasma levels observed from the bead and IV treatments when given 24 h apart and no difference between the pharmacokinetic profiles of the two cycles separated by 3 weeks. Irinotecan plasma AUC values were similar in both the embolization and IV arms of the study. C_max_ values obtained during the IV arms of the study are approximately double that of the embolization arms whilst T_max_ times are shorter in the IV arms, supporting extended release of drug from the beads. Bioavailability for bead-based delivery was double that for IV administration, which was attributed to reduced clearance of the drug when delivered by this route. No additive toxicity was observed as a consequence of the combined treatments. The combination of irinotecan delivery via drug eluting bead and IV was well-tolerated with no significant clinical effects. Pharmacokinetic analyses suggest the bioavailability from bead-based delivery of drug is double that of IV infusion, attributable to reduced drug clearance for the former.

## Introduction

Colorectal cancer is the third leading cause of death from cancer and the third most common malignancy in both men (after prostate and lung cancers) and women (after breast and lung cancers) in the United States. Upon diagnosis, metastasis of colorectal tumors is common, particularly to the liver which frequently induces patient death due to hepatic failure. Treatment options for patients with metastatic colorectal cancer (mCRC) are limited and clinical outcome is generally poor. Although surgical resection in selected patients can achieve 25–45 % 5-year survival, in all other patient groups it is less than 5 %. Systemic chemotherapy can palliate symptoms and improve survival and in recent years the topoisomerase I inhibitor irinotecan, has been approved for use in combination with 5-FU/folinic acid in patients without prior chemotherapy and for the second-line treatment of this disease as a single agent in patients who have failed an established 5-FU-containing treatment regimen [1].

There is increasing interest in local delivery of chemotherapy to the liver in an attempt to improve the effectiveness of these drugs against liver metastases [2]. Transarterial chemoembolization (TACE) has been used effectively in the local treatment of hepatocellular carcinoma (HCC) [3, 4] but in general, it is thought that it is the hypovascular nature of hepatic mCRC that renders them less suitable for treatment by conventional TACE [5]. Despite this, Phase II TACE studies for mCRC have been conducted in a number of centers, with one study reporting a complete response of 17 % and 1- and 2-year survival rates of 68 and 37 % respectively using doxorubicin and Lipiodol [6]. Others have reported a 63 % partial or minor tumor morphologic response, 62 % of patients with decreased carcinoembryonic antigen level greater than 50 % and a median survival of 10 months when treated by chemoembolization with 5-fluorouracil, mitomycin-C and gelatin sponge [7]. One of the largest series of patients to be treated to date consisted of 207 patients treated with repeated TACE at 4 week intervals using mitomycin C with/without gemcitabine and embolization using lipiodol and degradable starch microspheres [8]. Local tumor control yielded 12 % partial response, 51 % stable disease and 37 % progressive disease with 1- and 2-year survival rates of 62 and 38 % respectively. The investigators concluded that TACE is an effective minimally-invasive therapy for neoadjuvant, symptomatic or palliative treatment of liver metastases in colorectal patients.

DC Bead is an embolization device that has been shown to load and release drug molecules that carry a positive charge [9–11]. It has been used combined with doxorubicin in a number of studies for the treatment of HCC [12–15], cholangiocarcinoma [16] and neuroendocrine metastases [17]. It also has some interaction with certain campothecin analogues such as irinotecan [18] and topotecan hydrochloride [19, 20]. Preclinical studies have demonstrated that administration of DEBIRI in a porcine model of hepatic embolization is safe and results in a reduction of peak plasma drug levels compared to intrahepatic arterial or intravenous delivery of a bolus of irinotecan [21, 22]. Moreover, the beads have been shown to be efficacious in an embolization model of rat colorectal metastases [23]. Subsequent clinical evaluation has showed considerable early promise in the treatment of third line patients [24, 25] or patients refractory to systemic chemotherapy [26, 27]. Moreover, results from a small Phase III study showed a statistically significant improvement in survival (7 months), progression-free survival (3 months) and quality of life (5 months) for DEBIRI versus systemic treatment (intravenous FOLFIRI) [28]. These encouraging data have raised interest in treating first and second-line patients with DEBIRI, but in combination with other systemic agents to ensure treatment of non-hepatic disease [29]. The study presented here therefore aimed to gain information on the pharmacokinetics (PK), safety and tolerability of a regimen combining DEBIRI with intravenous (IV) systemic irinotecan in a well-published porcine model of hepatic arterial embolization. Two treatment cycles were performed, with up to either 100 or 200 mg of DEBIRI administered with IV irinotecan administered at either 250 or 350 mg/m^2^. During each treatment cycle the DEBIRI administration and the IV irinotecan therapy were administered on two consecutive days followed by a re-treatment with the combination therapy 3 weeks later.

## Materials and methods

### Overview of the study design

This study consisted of three groups of animals as summarized in Table 1 and carried out in accordance to EC Directive 86/609/EEC for animal experiments. In each group female domestic large white × landrace pigs were administered 100–300 μm Irinotecan Bead containing 100 mg drug/ml of lyophilized beads (Biocompatibles UK Ltd, Farnham, UK), via intrahepatic artery injection as a slow bolus, such that the test material was delivered until stasis was achieved or the maximum dose had been administered. This was followed by intravenous infusion of an irinotecan monotherapy (DBL^®^ Irinotecan Concentrate, Hospira Australia Pty Ltd, Mulgrave, Australia) over 30–90 min, 24 h post embolization. Each animal underwent two cycles of each treatment before necropsy of the group 1 animals at 6 weeks after initial embolization. The remaining four animals in groups 2 and 3 underwent necropsy 12 weeks after initial embolization.

**Table 1:** Overview of the study design

Group (*n*)	Test material	Irinotecan dose	Treatment regime	Dose volume	# Of cycles	Necropsy
1 (*n* *=* 3)	DEBIRI (100–300 μm)	To stasis (up to 100 mg)	Intrahepatic artery injection as a slow bolus	Up to 1 ml of beads (100 mg irinotecan/ml beads in a total volume up to 10 ml)	2	Week 6 ± 1 day after initial embolization
2 (*n* *=* 3)	Irinotecan mono-therapy	250 mg/m^2^	Intravenous infusion over 30–90 min 24 h post embolization	Final concentration range of 0.12–2.8 mg/ml administered in 250–500 ml of 0.9 % saline	2	Week 12 ± 1 day after initial embolization
3 (*n* *=* 1)	DEBIRI (100–300 μm)	To stasis (up to 200 mg)	Intrahepatic artery injection as a slow bolus	Up to 2 ml of beads (100 mg irinotecan/ml beads in a total volume up to 20 ml)	2	Week 12 ± 1 day after initial embolization
	Irinotecan mono-therapy	350 mg/m^2^	Intravenous infusion over 30–90 min 24 h post embolization	Final concentration range of 0.12–2.8 mg/ml administered in 250–500 ml of 0.9 % saline	2	

In group 1 and 2, the DEBIRI was administered up to a maximum of 100 mg irinotecan dose (using up to 1 ml of beads containing 100 mg irinotecan). Each vial of the beads was mixed with water for injection and contrast medium to a final volume of up to 10 ml. Irinotecan monotherapy was administered as an intravenous infusion of 250 mg/m^2^ dose over 30–90 min. The dose calculations for the irinotecan followed those in the literature [30], using the formula:

$$ {\text{Surface area}} = 0.0 7 3 4 \times \left( {{\text{weight}}^{0. 6 5 6} } \right) \times {\text{dose required}} $$

The solution was withdrawn from the vial and diluted in 0.9 % sodium chloride injection, BP, prior to infusion to a final concentration range of 0.12–2.8 mg/ml.

It was unknown at the time whether the animals would tolerate an escalated dosing regimen and hence just one animal was treated with higher drug concentrations (group 3). In this case, DEBIRI was administered up to a maximum of 200 mg irinotecan dose (using up to 2 ml of beads). Each vial of the beads was mixed with water for injection and contrast medium to a final volume of up to 10 ml. The irinotecan monotherapy was also escalated and administered as an intravenous infusion of 350 mg/m^2^ dose over 30–90 min.

Angiography was performed and recorded prior to, during and following DEBIRI administrations for verification of place location. At protocol-specified time points, clinical and vascular access site observations were performed, body weights were recorded, and blood samples were collected for evaluation of clinical pathology parameters. Additional blood samples were collected for quantification of irinotecan (CPT-11) and the metabolite SN-38. On day 42 (±1 day), the group 1 animals were humanely killed (or died) and subjected to a comprehensive necropsy. Samples were collected for histological assessment. On day 84 the remaining animals in groups 2 and 3 underwent these assessments.

### Interventional procedures

A cutdown procedure was used to isolate the jugular vein and two PVC catheters, with silastic tubing placed over the section of the catheter immediately proximal to the entry point, were inserted into the vein. Once the catheters were in the final position and had been checked to ensure that they were flowing well, they were sutured to surrounding muscle to stabilize the catheters in situ. The catheters were sutured to the skin (exposed) around the neck. The access site was sutured closed around the external region of the catheter. A zip-lock bag was sutured to the back of the animal and used to hold the catheters when not in use. One catheter was used for blood sampling, the other was used for the IV administration of irinotecan. The catheters remained in place up to 48 h post-infusion, after which they were removed. Subsequent blood samples were obtained by venepuncture of an appropriate vessel.

The femoral artery was accessed and an appropriately sized guidewire followed by an introducer “portal entry” then a 4F guide catheter were advanced into the common hepatic artery. The catheter was passed over the guidewire into the left liver lobe and the guidewire was subsequently removed. Angiography was performed to confirm the catheter position in the target region. With the catheter in position, up to 2 ml of DEBIRI (in a total volume of up to 20 ml) were slowly administered, at a rate to minimize reflux from the target area. The total volume administered was recorded (Table 2). A final angiogram was performed 5 min after delivery of the microspheres was completed. After the final angiogram, the catheter and introducer sheath were removed from the animals. The femoral artery was ligated or pressure applied to the access site until haemostasis was obtained and the site then sutured closed. Output angiograms were used to qualitatively assess the extent of embolization. The angiograms were taken prior to embolization, during embolization and 5 min post-embolization. Where complete embolization was judged to have occurred, the artery antegrade to the tip of the catheter displayed as a blind vessel with essentially no flow, although some contrast was present still in the lobe. Scores were graded by comparing the speed of antegrade flow within the liver compared to the speed of antegrade flow within non-embolized vessels. Reflux of contrast media in a retrograde direction after injection was also considered when assessing embolization. Vascular flow was thus graded from zero (complete occlusion) to three (no occlusion).

**Table 2:** DEBIRI dose administered and extent of embolization

	Phase 1 (day 1)			Phase 2 (day 22)				
Group	Irinotecan (mg)	Stasis (Y/N)	Score (0–3)	Irinotecan (mg)	Stasis (Y/N)	Score (0–3)	Score final	Comment
1	100	N	1	100	N	2	2	Close to stasis
1	100	Y	0	100	N	1	2	
1	100	Y	0	100	Y	0	–	No score—died
2	100	N	1	100	N	2	2	
2	100	N	1	100	N	2	2	
2	100	N	2	100	N	2	2	
3	200	Y	0	135	Y	0	1	Not all beads in

The intravenous administration of irinotecan was performed on conscious animals. The infusion was administered via a previously placed jugular catheter. The drip rate was set to approximately 160 drops/min. The animals received the appropriate volume of irinotecan in 500 ml of sterile 0.9 % saline. Heart rate (HR), oxygen saturation and other parameters were measured by pulse oximetry.

Individual dose administration data and extent of embolization are presented in Table 2. DEBIRI was administered to the left lobe of the liver. All animals in groups 1 and 2 received their full dose of DEBIRI (100 mg) on both occasions. The group 3 pig received the full 200 mg at the primary embolization, but only 135 mg on the secondary embolization due to stasis being achieved at that point. Table 3 shows the amount of irinotecan administered intravenously on the two occasions. All animals received their full dose of irinotecan. There were instances of frothing at the mouth or coughing in all pigs.

**Table 3:** Doses of IV irinotecan administered

		Phase 1 (day 2)			Phase 2 (day 23)		
Group	Dose (mg/m^2^)	Weight (kg)	Irinotecan (mg)	Irinotecan (ml)	Weight (kg)	Irinotecan (mg)	Irinotecan (ml)
1	250	31.0	174.6	8.7	37.5	197.8	9.9
1	250	31.0	174.6	8.7	40.0	206.3	10.3
1	250	30.0	170.9	8.5	36.5	194.3	9.7
2	250	32.0	178.2	8.9	45.5	224.5	11.2
2	250	33.5	183.7	9.2	43.5	218.0	10.9
2	250	32.5	180.1	9.0	39.5	204.6	10.2
3	350	30.5	241.8	12.1	38.5	281.7	14.1

### Blood sample collection and processing

Blood samples for hematological and biochemical analyses were collected prior to embolization (baseline), and on days 2, 3, 7, 14, 22 (prior to embolization), 23, 24, 28, 42, 63 and 84 (all collections after day 3 are ± 2 days). Samples for pharmacokinetic analyses were collected prior to embolization (baseline), immediately on completion of embolization (0 time, post-embolization) and at 2, 5, 10, 15, 30, 60, 90 min, 2, 3, 4, 6, 9, 12, 16, 20 h following completion of embolization; and prior to IV infusion (24 h post-embolization), 2, 5, 10, 15, 30, 60, 90 min, 2, 3, 4, 6, 9, 12, 16, 20, 24 and 48 h post-infusion. For haematology measurements, blood samples were collected into tubes containing tri-potassium (K_3_)EDTA and were analysed for the laboratory standard complete blood examination (CBE) parameters using a SYSMEX-XE-2100 (TOA Medical Electronics, Kobe, Japan) hematology analyzer. A blood film was analyzed for the white cell differential and any abnormal cells. For biochemistry measurements, blood samples were collected into tubes containing a clot activator and were processed for serum. The standard multiple biochemical analysis (MBA) parameters and lipid screen were determined using an Olympus AU5400 (Olympus America Inc, NY, USA) chemistry analyzer. Whole blood samples for pharmacokinetics bioanalysis were collected into tubes containing lithium heparin, centrifuged for 5 min at 3000 rpm and the plasma was extracted. The plasma was immediately stored at −20 °C. The frozen plasma samples were analyzed for irinotecan and the metabolite SN-38 using a validated HPLC method (Shimadzu Corporation, Kyoto, Japan).

### Necropsy, tissue collection and histopathology

All pigs were subjected to a comprehensive necropsy, and any macroscopic abnormalities were noted. Sections of liver (site of embolization and non-embolized liver tissue), duodenum, jejunum, ileum, colon, caecum, stomach, kidneys, gall bladder, spleen, lung, heart and brain were dissected free and fixed in 10 % neutral buffered formalin. Tissue samples collected on day 42 ± 1, and day 84 ± 1, were trimmed, embedded, and sectioned. The slides were stained with hematoxylin and eosin.

### Pharmacokinetics

The following parameters were derived or calculated where possible for both CPT-11 and SN-38; area under the concentration–time curve (AUC), half-life, T_max_, and C_max_. The T_max_ and C_max_ values were obtained by visual examination of the data. The AUC values were calculated using the trapezoid rule by GraphPad Prism 4 (GraphPad Software Inc, CA, USA). The terminal elimination half-life (t_1/2_) was determined by linear regression of the terminal log-concentration data points after plotting the plasma concentrations against time, using the formula:

$$ T1/2 = \frac{0.693}{\beta } $$

where β = slope × (–2.303).

In order to make estimates of clearance and bioavailability of the drug, the AUCs were extrapolated to infinite time and then normalized to 174 mg dose (equivalent to 250 mg/m^2^ dose in a nominal typical animal), giving nAUC_0−inf_. The bioavailability (F) was calculated as the ratio of the nAUC_0−inf_ values for bead-based to IV delivery routes. Clearance was calculated as the absolute dose administered divided by AUC_0−inf_, resulting in systemic clearance (CL) for IV and apparent clearance (CL/F) for bead-based delivery. All PK parameters in the text are reported as mean ± standard deviations (SD).

## Results

### Body weight

**Fig. 1:**
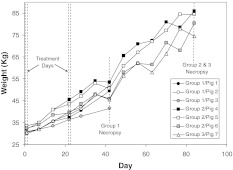
Body weight of pigs over the experimental period (*note*: animals were non fasted on days 35, 49, 56, 70 and 77. All other weights are of fasted animals)

Changes in body weights are shown in Fig. 1. Body weights at the time of the initial intervention ranged from 30.0 to 33.5 kg. Body weight increased in all animals over the duration of the study, although one animal (pig 3) did not show the same degree of increase as the others, was moribund on day 41, and died during the final angiogram. This was a consequence of a generalized infection and was not treatment-related.

### Hematology

**Fig. 2:**
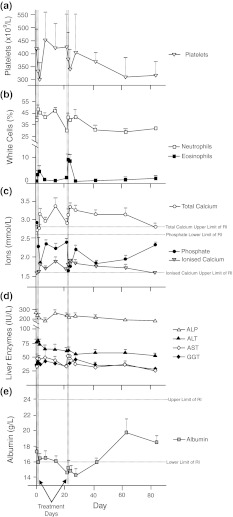
Hematological and biochemical parameters (+SD, *n* *=* 7, 1–42 days; *n* *=* 4, 43–48 days): **a** Mean platelets; instances of clumped platelets have been excluded. **b** Mean neutrophil and eosinophil % of total white cell counts. **c** Mean phosphate, total calcium and ionized calcium (calculated). **d** Mean concentrations of gamma glutamyltransferase (*GGT*), alkaline phosphatase (*ALP*), alanine aminotransferase (*ALT*) and aspartate aminotransferase (*AST*). **e** Mean serum albumin concentration

The data for groups 1 and 2 up to day 42 were compared to the baseline (pre-embolization) values. The group 3 animal was excluded from these analyses. The only statistically significant changes from baseline were in the platelet counts at days 2 (*P* *<* 0.001), 3 (*P* *<* 0.001), 23 (*P* *<* 0.01), 24 (*P* *<* 0.001) and 28 (*P* *<* 0.01) and 42 (*P* *<* 0.001). On each occasion, the values were lower than the initial mean platelet count. There was a wide variation in platelet counts between the pigs (see Fig. 2), with only one animal having platelet counts outside the reference interval (>550 × 10^9^/l). The data for the group 2 animals at days 63 and 84 were also compared to baseline. Again, the only significant differences were in the platelet counts (*P* *<* 0.01 on both days).

Eosinophil counts increased in all animals except one on day 23, and remained high on day 24 in five animals. The eosinophils were also increased from baseline on days 2 or 3 in three animals. There was a transient increase in the mean (*n* = 7) neutrophil and eosinophil counts in the 48 h following treatments, which returned to baseline. These are shown in Fig. 2. There was no evidence of neutropaenia, which has been reported in human studies of various dosing regimens [31].

### Biochemistry

The data for the group 1 and 2 animals up to day 42 were compared to the baseline (pre-embolization) values. The only statistically significant differences from baseline were on day 2 (LDH increased, *P* *<* 0.001); day 3 (LDH increased, *P* *<* 0.05); day 7 (ALP decreased, *P* *<* 0.05); day 14 (LDH increased, *P* *<* 0.001); day 23 (LDH increased, *P* *<* 0.001). The data for the group 2 animals for days 63 and 84 were also compared to baseline. Again, the only significant differences were in the LDH (*P* *<* 0.0001) on day 63. Following treatments, there were increases in mean (*n* *=* 7) serum total and ionised calcium levels, and decreases in serum phosphate (Fig. 2). The elevated serum calcium concentrations were not observed in a previously reported study where the pigs only had one intervention [21]. These findings were considered not clinically significant. Changes in mean enzyme levels were minor, with AST showing an increase following treatments (Fig. 2). There was a slight decrease in the mean serum albumin concentrations (*n* *=* 7) following the initial treatments (still within reference interval to day 22), and these remained decreased to day 28 (below reference interval). The total protein concentrations were within the reference interval throughout.

### Plasma pharmacokinetics

**Fig. 3:**
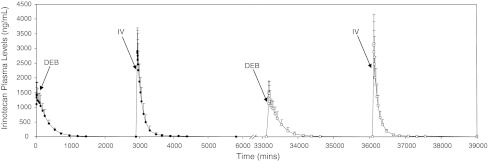
Plasma level profiles of irinotecan from both cycles of bead-based and IV delivery, demonstrating no overlap of systemic drug from the two treatments in either cycle

**Fig. 4:**
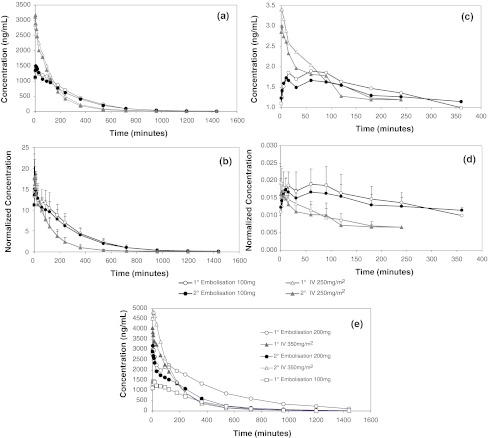
Plasma level profiles for primary and secondary cycles: **a** irinotecan 100 mg DEB and 250 mg/m^2^ IV; **b** irinotecan 100 mg DEB and 250 mg/m^2^ IV normalized for dose; **c** SN-38 100 mg DEB and 250 mg/m^2^ IV; **d** SN-38 100 mg DEB and 250 mg/m^2^ IV normalized for dose; **e** irinotecan 100 mg and 200 mg DEB and 350 mg/m^2^ IV

All pre-embolization samples were less than the lower limit of quantification (LOQ) for both analytes. Figure 3 depicts the average irinotecan plasma profiles for following DEBIRI and IV irinotecan administration in groups 1 and 2. The overall profiles for the two cycles appear similar, and following bead delivery, the levels of the drug in the plasma drop below the LOQ before the IV dose is given. Figure 4 shows the actual irinotecan levels for each cycle (Fig. 4a, error bars removed for clarity) with the same data in which the concentrations have been dose-normalized for comparative purposes (Fig. 4b) and the same graphs for SN-38 (Fig. 4c, d). Clearly, from Fig. 4b the dose-normalized AUC values for the bead-based delivery are substantially higher than for IV delivery (from Table 4, 12821.7 and 11838.9 ng h/ml for the primary and secondary bead delivery versus 6330.8 and 5427.6 ng h/ml for the primary and secondary IV delivery). This results in a bioavailability (F) for bead-based delivery of 206 and 220 % for the primary and secondary interventions respectively. As it is not possible to deliver more dose than that administered, this increase in F is attributed to a reduced clearance for bead-based delivery of irinotecan (Table 4). Figure 4e shows the irinotecan plasma levels for the group 3 animal with escalated dose. This figure also includes the curve of the primary embolization with DEBIRI at 100 mg dose to allow comparison between 200, 135 and 100 mg DEBIRI doses. Although these curves have not been dose-normalized, it is clear the significant impact the increased dose has on the AUC.

**Table 4:** Pharmacokinetic parameters for irinotecan delivery

Phase	Parameter	Average	SD	CV (%)	Units
1° Embolization	Dose	100	0	0	mg
	k	0.219	0.026	12.09	/h
	AUC_0−t_	7307.10	2129.28	29.14	ng h/ml
	nAUC_0−inf_	12821.72	3714.94	28.97	ng h/ml
	CL/F	240.22	60.38	25.13	ml/min
	F	205.54	18.72	9.11	%
2° Embolization	Dose	100	0	0	mg
	k	0.204	0.038	18.62	/h
	AUC_0−t_	6724.38	2351.96	34.98	ng h/ml
	nAUC_0−inf_	11838.92	4130.29	34.89	ng h/ml
	CL/F	266.41	75.94	28.51	ml/min
	F	219.06	22.85	10.43	%
1° IV	Dose	177.02	4.58	2.58	mg
	k	0.112	0.016	14.04	/h
	AUC_0−t_	6345.13	2374.00	37.41	ng h/ml
	nAUC_0−inf_	6330.76	2264.09	35.76	ng h/ml
	CL	494.76	127.70	25.81	ml/min
2° IV	Dose	207.58	11.64	5.61	mg
	k	0.143	0.044	30.54	/h
	AUC_0-t_	6309.65	2047.31	32.45	ng h/ml
	nAUC_0−inf_	5427.57	1909.68	35.18	ng h/ml
	CL	580.44	162.27	27.96	ml/min

The mean PK data (±SD) for irinotecan and SN-38 measured in each phase of the study for groups 1 and 2 are shown in Table 5. Data from the group 3 animal are omitted from the tables due to the higher dose of irinotecan that was given to this pig. The terminal elimination half-life values for SN-38 were not calculated for some profiles due to insufficient or non-conforming data. In general, SN-38 plasma concentrations were very low (about 1,000 times lower than irinotecan) and were either just above or below quantification limits in most cases.

**Table 5:** Key pharmacokinetic data

Phase	AUC (ng/ml h)	T_½_ (h)	C_max_ (ng/ml)	T_max_ (min)
Irinotecan				
1° Embolization	7307 ± 2129	3.2 ± 0.4	1493 ± 432	10.0 ± 4.5
2° Embolization	6724 ± 2352	3.5 ± 0.8	1571 ± 396	6.2 ± 4.5
1° IV	6345 ± 2374	6.3 ± 0.9	2837 ± 709	0.7 ± 1.0
2° IV	6310 ± 2047	5.4 ± 2.0	3302 ± 840	0.7 ± 1.0
SN-38				
1° Embolization	5.5 ± 3.6	5.0 ± 3.6	1.7 ± 0.9	26.0 ± 20.4
2° Embolization	5.9 ± 2.9	5.9 ± 2.9	1.9 ± 0.2	43.3 ± 45.8
1° IV	5.0 ± 2.1	2.0 ± 0.5	3.5 ± 0.8	1.2 ± 2.0
2° IV	4.4 ± 2.0	2.2 ± 0.3	3.1 ± 0.5	1.0 ± 1.1

Initial inspection of the IV data indicates clear 2-compartment pharmacokinetics, demonstrated as an initial rapid first order (i.e. linear on a log scale) decline in concentrations until approximately 500–750 min, presumably due to distribution of irinotecan out of the blood into tissues (the “distribution phase”). This is then followed by a slower “terminal” phase, presumably due to elimination of the drug. The terminal phase appears to be first-order from 900 min onwards. Examination of the DEBIRI curves reveals a slightly more complex plasma profile, for which there is a tendency for an initial decline in levels during the first minutes, followed by a small plateau or elevation for a few hours and subsequently a slower decline from thereon (best seen in Fig. 4e for the primary embolization using the 200 mg dose). After some hours there becomes a much slower mono-exponential (1-compartment) decline. This also appears to be much slower than the initial distribution-phase decline in concentrations in comparison to the IV data but closer inspection reveals some evidence that there may be a second slower phase which occurs at the very end of the study.

### Histopathology

**Fig. 5:**
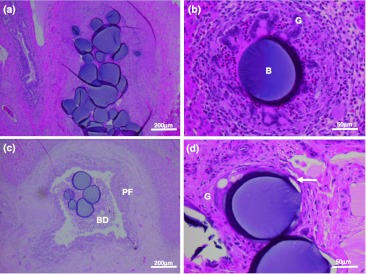
Selected histopathology: **a** numerous beads in a hepatic artery; **b** bead (B) surrounded by a granulamatous inflammatory reaction of epithelioid macrophages and multinucleated giant cells (*G*), interspersed with eosinophils; **c** bead in the lumen of a bile duct (*BD*); there is also marked periportal fibrosis (*PF*); **d** beads in hepatic arteries with marked attenuation of the vessel wall (*arrow*) (necrotizing vasculopathy) and early granulomatous response (*G*)

Microscopically, in the liver of all pigs, morphological changes resulting from bead injection were principally characterized by a necrotizing vasculopathy in portal arteries, and a granulomatous inflammatory reaction surrounding the beads, with substantial portal fibrosis and inflammatory infiltration. This is typical of what has been previously reported [21]. A variable number of beads were found in hepatic portal tracts, ranging from one to up to ten (Fig. 5a), which is a factor of the vessel size. Beads were frequently found in the lumina of degenerate blood vessels, often surrounded by a granulomatous reaction of epithelioid macrophages and multinucleated giant cells (consistent with the expected foreign body response), (Fig. 5b), or occasionally in the lumen of bile ducts (Fig. 5c).

There were many similarities between the hepatic pathology in the groups 2 and 3 pigs at 84 days and the group 1 pigs at 42 days. Hepatic arteries containing beads in the portal region showed a necrotising vasculopathy, often surrounded by a granulomatous reaction or concentric fibrosis. There was marked periportal fibrosis with encroachment upon, and replacement of, contiguous hepatocytes and a generally mild mononuclear cell infiltrate sometimes with a few eosinophils. On occasion in two animals there was marked bile ductule hyperplasia. Lymphoid nodules were occasionally found in the portal region and a few blood vessels sometimes showed marked smooth muscle hypertrophy. In many livers, there was mild, multifocal hepatocyte necrosis with a mononuclear cell reaction, including Kupffer cells. Thrombi in hepatic vessels were not found, except in the group 3 pig in the left lateral lobe, which produced an infarct. There was an occasional bead found in a bile duct lumen and in gastric and duodenal walls, while beads were frequently found in mesenteric blood vessels of the porta hepatis. These findings were more common in the group 3 pig that had received the largest volume of beads. The main histological differences between this study and that previously reported [21] were the higher frequency of lymphatic dilatation in the current study, and the reduced incidence of hepatic vessel thrombi, with the exception of the group 3 pig which received the higher dose and hence volume of beads.

While the hepatic arteries containing beads were usually recognisable as blood vessels, lumina were markedly dilated and walls invariably attenuated and generally represented by a narrow band containing pyknotic myocyte and endothelial nuclei. Affected vessels were surrounded, to varying degrees, by a granulomatous inflammatory reaction and fibroplasia. This total or subtotal destruction of vessel walls, putatively due to pressure atrophy from intraluminal beads, was designated a necrotizing vasculopathy (Fig. 5d). Periportal fibrosis (Fig. 5c) was usually moderate to severe with encroachment upon, and concomitant loss of, surrounding hepatic parenchyma and there was invariably an inflammatory infiltrate comprised largely of lymphocytes and eosinophils.

In order to analyze the lobar distribution of injected beads, lobes were blind coded for histopathological examination and six sections of approximately equal size randomly selected from each lobe. The number of beads in each section was then counted and summed for each lobe. In all pigs beads were preferentially located in the left hepatic lobes (lateral and central) although for one pig in group 1, numerous beads were also found in the right lateral lobe, indicating an off-target delivery. In the group 3 pig, there were more beads in the caudate lobe than for any of the other pigs, a result of the increased volume administered. The number of beads found in other lobes was small.

## Discussion

The treatment options for metastatic colorectal cancer to the liver have broadened in recent years, with the advent of more effective agents such as irinotecan and oxaliplatin resulting in an increase in median survival from 6- to over 20 months [32]. The combination of irinotecan with bolus 5-fluoruracil and leucovorin (IFL) with bevacizumab (an antibody that targets vascular endothelial growth factor) offers further advantage as a first line therapy [33]; whilst irinotecan-refractory patients show improved response and longer median time to progression when combined with cetuximab, an antibody targeting epidermal growth factor receptor [34]. All of these treatments however, have associated dose-limiting toxicity, with frequent mucositis, nausea and vomiting, alopecia and in some cases severe diarrhea. Local therapy of colorectal metastases to the liver using DEBIRI in third-line patients has resulted in reported reductions in some of these side-effects [24]. There is therefore mounting interest in combining systemic and local-based therapy of tumours, one aim being to attempt to reduce the total systemic drug load and hence limit toxicity. Moreover, tumor spread can be controlled via the combined embolic effect and local delivery of chemotherapy attained using drug eluting beads. This type of multi-modal therapy could potentially present issues with overlapping toxicities or prolonged and/or cumulative effects that have not been previously observed in vivo. This preclinical study was therefore designed to test the feasibility of performing TACE with DEBIRI followed by IV irinotecan at clinically-relevant doses, to monitor the safety of the procedure and examine the pharmacokinetics of the drug from the two different routes of administration.

In terms of the effect of the combined procedure on the liver function of the animals, statistically significant changes from baseline in clinical pathology (hematology and serum chemistry) were seen only in platelet counts, LDH and ALP. It has been previously observed however, in human studies involving hepatic TACE, that it is not unusual for many liver enzyme levels to be elevated five to 20-fold post-procedure [35, 36]. In this context, the changes seen here are minor and unlikely to be of concern with respect to clinical translation.

Irinotecan plasma AUC values are very similar in both the embolization and IV arms of the study. Allowing for dose-normalization, the bioavailability for bead-based delivery increases to an average of 210 %, attributable to a decreased drug clearance for this route of administration. Hepatic clearance is determined by liver blood flow, intrinistic metabolic clearance and the unbound fraction of drug in blood. It is likely all three factors contribute to the reduced clearance, but in particular the first two mechanisms, as the embolic beads will obstruct arterial blood flow and local delivery of high concentrations of drug into the liver parenchyma could potentially saturate enzymes responsible for the drug’s metabolism. C_max_ values obtained during the IV arms of the study are approximately double that of the embolization arms, whilst T_max_ times are shorter, both supporting the premise of slower release of the drug from the beads compared to the IV infusion. This study did not determine the form of the irinotecan, as it is known to exist in its active lactone and less active carboxylate forms, the ratio of which is determined by a pH driven equilibrium. It has been demonstrated however, that it is the active lactone form of the drug which exists within the beads and which is continually eluted at the site of delivery [37].

The shape of the PK plasma profiles for the IV and bead administrations differ significantly. If attempts are made to model the bead PK profile, the most appropriate fit to the data assumes an immediate bolus of drug followed by a much slower infusion over the ensuing hours; this leads to slightly elevated plasma levels of drug and metabolite during this phase in comparison to IV administration when normalized (Fig. 4c, d). This initial burst of drug can be rationalized, as depending upon the type of contrast agent used to suspend the beads and visualize delivery, some 5–15 % of the loaded irinotecan dose can be displaced into the suspension medium upon mixing and hence would effectively provide an immediate intra-arterial bolus. This is then followed by the slower release phase of drug elution from the beads, which may give rise to a ‘bump’ in the PK profile when the rate of infusion is close to or faster than the rate of clearance.

T_½_ appears longer in the IV arms compared to the embolization arm, which is an unexpected discrepancy given the evidence for reduced clearance of drug after bead-based delivery, which should yield a longer T_½_. The apparent mono-exponential (1-compartment) decline of drug after bead-based delivery is slower than the initial distribution-phase decline for IV but there is evidence for a second slower phase occurring at the end of the sampling period (between 900 and 1,500 min), seen as a change in slope of the decline. This is most probably the consequence of “flip-flop” kinetics typically observed for sustained release systems, in which the absorption rate (i.e. composite of the release of irinotecan from the beads, diffusion through tissue and its subsequent appearance in the blood) becomes rate-limiting over the elimination process [38].

SN-38 plasma AUC values are approximately 1,000-fold less than those obtained for irinotecan. SN-38 plasma AUC values are similar in both the embolization and IV arms of the study. C_max_ values do appear to be higher, and T_max_ values shorter, during the IV arm of the study than those obtained from the embolization arm but again, dose-normalization suggests the bead-based delivery may afford a longer phase of low-levels SN-38 exposure. This could be advantageous, as there are reports in the literature that prolonged exposure to low levels of SN-38 afford greater anti-tumor activity [39]. SN-38 has been implicated in the appearance of late-onset diarrhea, which is difficult to treat and is thought to be associated with SN-38-induced GI mucosal toxicity [40]. Interestingly, in studies performed to date with DEBIRI, no significant side effects related to diarrhea have been reported [24, 25, 41, 42].

From a pharmacokinetic stand-point there appears to be no difference between the primary and secondary phases of the study, other than for the group 3 animal, the entire 200 mg dose could not be administered in the second cycle and hence the plasma levels were correspondingly lower. At 24 h post embolization for both cycles of treatment, the drug had clearly dropped below the LOQ and there was therefore no overlap in the plasma levels with the IV administration and hence risk of over-dosing due to overlapping toxicities.

At necropsy, one animal had a generalized infection with tissue changes (unrelated to treatment regimen), and another animal had an area of pallor in the left lateral liver lobe (an infarct due to arterial thrombosis). Microscopically, in the liver of all pigs, morphological changes resulting from bead injection were principally characterized by a necrotizing vasculopathy in portal arteries, and a granulomatous inflammatory reaction surrounding the beads, with substantial portal fibrosis and inflammatory infiltration. These observations were the same as those typically observed in the same animal model, in which animals were treated with DEBIRI on their own [21]. The more aggressive treatment regimen followed in this study also largely compared to that previously reported, resulting in only minor differences between the two. In the current study there was an increase in serum calcium concentrations not seen in the previous study. Histologically, there was a higher frequency of lymphatic dilatation in the current study compared to previously, but a reduced incidence of hepatic vessel thrombi, with the exception of the group 3 pig which received the higher dose. Overall, there were no findings that would suggest any undue additive toxicity was experienced by combining bead-based therapy with IV delivery of irinotecan.

## Conclusion

Embolization using a 100 mg dose of DEBIRI, followed by IV administration 24 h later at 250 mg/m^2^ was a well-tolerated treatment regimen, with only minor changes in hematological and biochemical parameters which were not clinically significant. The more aggressive treatment regimen of the group 3 animal showed similar results to those in groups 1 and 2, with no apparent additional toxicity which suggests the use of even higher doses could be possible.

There was evidence that the bioavailability of irinotecan from hepatically implanted beads in the pig is an average of 200–220 %. This apparent doubling of the systemic availability is a physical manifestation of the reduced systemic clearance of the drug. This is both a consequence of the primary mode of action of the embolic device to reduce hepatic blood flow and to a decrease in the intrinsic metabolism of the drug due to local delivery from the device. The clinical importance of these unique pharmacokinetic aspects of bead-based delivery may be that (i) similar systemic exposure to irinotecan is possible using doses that are approximately 50 % smaller than IV administration, (ii) potentially greater localized exposure of the liver may be obtained which may afford a greater effect on hepatic tumors.

This study therefore indicates it is feasible to undertake a combination of systemic and bead-based delivery of irinotecan without fear of over-dosing the systemic circulation as long as the embolization and IV infusion are separated by at least a 24 h period. Liver function and histopathological changes are not significantly affected over that seen for embolization alone. Further studies are on-going to provide further valuable insight into the potential of this novel method of treatment of colorectal metastases to the liver.

## Abbreviations

TACE: Transarterial chemoembolization
mCRC: Colorectal cancer metastases
DEBIRI: Drug-eluting beads loaded with irinotecan
